# Heme-oxygenase-1 (HO-1) in CCR2/CCL2/CD200 brain mononuclear cells contribute to neuroinflammation in young adulthood after chorioamnionitis

**DOI:** 10.21203/rs.3.rs-10769119/v1

**Published:** 2026-10-19

**Authors:** Phoebe Kim, Nicholas Wolanske, Milind Kunale, Shenandoah Robinson, Lauren Jantzie, Maide Ozen

## Abstract

Chemokine receptor 2 ^+^ (CCR2 ^+^ ) mediates peripheral mononuclear cell infiltration into the brain via its ligand C motif ligand 2 (CCL2), whereas Immune inhibitory ligand CD200, suppresses mononuclear cell activation. Whether sustained alterations in CCR2/CCL2/CD200-expressing mononuclear cell populations and their Heme-oxygenase-1 (HO-1) expression contribute to persistent neuroinflammation following perinatal chorioamnionitis (CHORIO) remains unknown. We hypothesized that HO-1 expression within CCR2/CCL2/CD200 mononuclear cell subsets is altered in adulthood following perinatal CHORIO compared with sham controls. Brains were collected at postnatal day (P) 21 and P60 (n = 6–8/group), dissociated and surface and intracellular markers used to identify inflammatory and oxidative mononuclear cell subsets with flow cytometry. At P60, CHORIO brains exhibited fewer CCR2 ^+^ CD11bc ^L/INT^ cells (p = 0.008), increased intracellular HO-1 expression, (p = 0.01) and a reciprocal increase in CCR2 ^-^ CD11bc ^L/INT^ cells (p = 0.008). In addition, CCL2 ^+^ /CCR2 ^+^ CD11bc ^L/INT^ (p = 0.04) and CD200 ^+^ /CCR2 ^+^ CD11bc ^L/INT^ (p = 0.005) ratios were also increased in CHORIO. HO-1 expression in CCR2 ^+^ CD11bc ^L/INT^ cells was lower at P21 than at P60, suggesting an age-dependent phenotypic shift after CHORIO. Here we demonstrate that alterations in CCR2/CCL2/CD200 signaling and HO-1 expression in adult brain mononuclear cells following perinatal CHORIO are sustained, suggesting long-term inflammatory and oxidative immune signaling that may contribute to chronic neuroinflammation.

